# Tragedy in moral case deliberation

**DOI:** 10.1007/s11019-016-9749-7

**Published:** 2016-12-02

**Authors:** Benita Spronk, Margreet Stolper, Guy Widdershoven

**Affiliations:** 1 VU Medisch Centrum, De Boelelaan 1117, Postbus 7057, 1007 MB Amsterdam, The Netherlands; 2 Metamedica, De Boelelaan 1089 a, F-vleugel/Medische Faculteit, 1081 HV Amsterdam, The Netherlands

**Keywords:** Tragedy, Moral case deliberation, MCD, Decision-making, Dilemma method, Contingency

## Abstract

In healthcare practice, care providers are confronted with tragic situations, in which they are expected to make choices and decisions that can have far-reaching consequences. This article investigates the role of moral case deliberation (MCD) in dealing with tragic situations. It focuses on experiences of care givers involved in the treatment of a pregnant woman with a brain tumour, and their evaluation of a series of MCD meetings in which the dilemmas around care were discussed. The study was qualitative, focusing on the views and experiences of the participants. A case study design is used by conducting semi-structured interviews (N = 10) with health care professionals who both played a role in the treatment of the patient and attended the MCD. The results show that MCD helps people to deal with tragic situations. An important element of MCD in this respect is making explicit the dilemma and the damage, demonstrating that there is no simple solution. MCD prompts participants to formulate and share personal experiences with one another and thus helps to create a shared perception of the situation as tragic. The article concludes that MCD contributes to the sharing of tragic experiences, and fosters mutual interaction during a tragedy. Its value could be increased through explicit reflection on the aspect of contingency that characterises tragedy.

## Introduction

In professional practice, care providers are confronted with tragic situations, in which they are expected to make choices and decisions that can have far-reaching consequences. The dilemmas faced by practitioners are often urgent, requiring immediate decision-making. Frequently the choice to be made is not between good and evil, but between a greater and a lesser evil. Should a practitioner proceed with an operation that will extend the patient’s life by only a few weeks? Or is it preferable to withhold treatment, to ensure better quality of life? Should artificial respiration be given to a severely disabled infant with bleak prospects, or should the focus be on keeping the child comfortable and reducing suffering? The choice is between two evils, and searching for the best treatment option.^1^ Although care providers must make decisions regarding what medical action to take, this does nothing to lessen the tragedy of the situations they face. Every option has an inevitable moral downside.

To support care providers in making these choices, many Dutch hospitals offer ‘ethics support’. Research by Dauwerse et al. (2011, 84) has shown that 81 per cent of Dutch healthcare institutions acknowledge the necessity of clinical ethics support, stating its purpose as ‘promoting decisions with an ethical dimension’. Moral case deliberation is one of the instruments used as part of clinical ethics support (Dauwerse 2014). During moral case deliberation, healthcare professionals use a concrete case to explore what is at stake in a moral dilemma, and to identify the associated key (and possibly conflicting) values.

This article discusses an instance of moral case deliberation in a case that the participants clearly identified as tragic. Tragedy has many forms. On the one hand there is the tragedy as experienced by the patient. The case in question involved a pregnant woman with a brain tumour. She suffered severe from her illness and stood before the choice of keeping hope or accepting the end. On the other hand there is the tragedy as experienced by the care providers standing at her side, supporting her in the choices she had to make, feeling responsible for the decisions they had to take. This article focusses on tragic as experienced by care providers. Interviews were conducted with those involved to determine what it was that made the situation tragic for them, and how the inherent tragedy was discussed during the deliberations. The central question addressed by this study is: What is the role of moral case deliberation in dealing with tragic situations?

We will begin by defining the concept of tragedy based on literature. This is followed by an introduction of moral case deliberation as an instrument to support healthcare professionals in dealing with ethical issues. After that, we will describe the research method used. Next the results of the study are presented, ordered according to three sub-questions: What characterises this case as tragic? How does moral case deliberation bring this tragedy into focus? What do people need in tragic situations? The discussion analyses the findings, and concludes that moral case deliberation contributes to the sharing of tragic experiences, and aids mutual interaction during a tragedy. Its value could be increased through explicit reflection on the aspect of contingency that characterises tragedy.

## The concept of tragedy

People cannot control life. Things will always happen that we are powerless to change. This idea is given profound expression in the Greek tragedies, which examine the attempts made by people to come to terms with the things that happen to them: with undeserved setbacks, violence or the irrevocable nature of events (Manschot 2003, 226). Tragedy also relates to the vulnerability of life (Nussbaum 2001a, 399), and if there is any place where life’s vulnerability is patently evident, it is a hospital. Patients are confronted with the vulnerability of their own bodies, and—through their patients—practitioners encounter vulnerability in the form of the realisation that not all illnesses can be cured. As autonomous agents, this is hard to bear. We would rather be immune to setbacks (Sloterdijk 2004, 192 v, 249, 534). But where the real challenge lies, according to Nussbaum, is in tragic conflict. ‘In such cases we see a wrong action committed without any direct physical compulsion and in full knowledge of its nature, by a person whose ethical character or commitments would otherwise dispose him to reject the act. The constraint comes from the presence of circumstances that prevent the adequate fulfilment of two valid ethical claims. Tragedy tends, on the whole, to take such situations very seriously. It treats them as real cases of wrong-doing that are of relevance for an assessment of the agent’s ethical life.’ (Nussbaum 2001a, 25). As an example Nussbaum cites Agamemnon, who must sacrifice his daughter in order to save the expedition he is leading. He must choose, and is consumed by the impact of the decision he is to make. ‘He acknowledges that there is wrong done whichever way he chooses’ (Nussbaum 2001a, 35). The gods have put him in this situation, and there is no blameless escape (34). Nevertheless, Agamemnon still sees it as his own decision for which he himself bears responsibility (35).^2^

In another article (Nussbaum 2000, 1005–1036), Nussbaum draws a distinction between situations in which one must decide on a course of action (‘the obvious question’) and where a cost-benefit analysis can be applied, and situations involving the question of what one must give up (‘the tragic question’). The latter case involves a dilemma—two alternatives that both result in a loss and are morally objectionable (Molewijk et al. 2008; Stolper et al. 2016). A cost-benefit analysis is of no use in such cases. The question is more than a mere study of how to best consider the available courses of action—it concerns the limitations of such considerations, and the understanding that weighing up options does not help one to decide what constitutes a good life (Manschot 2003, 237).

This article is based on Nussbaum’s definition of tragic conflict. A tragic situation is one in which one is forced to make a choice that will inevitably be accompanied by moral objections. Tragedy and dilemma go hand in hand. Healthcare professionals play a role as actors in the tragic dilemma. This highlights the importance of tragic casuistry and demonstrates that for healthcare professionals, there is much at stake.

Confrontations with tragedy are not necessarily always negative experiences. Life is also enriched by the fact that we can be touched by others, and by what we experience. Friendship and love may bring vulnerability to life, but they are also precisely what give it value. By holding these values high, we also render ourselves vulnerable to potential threats. Although it may be our deepest wish to control or resolve tragedy, it is important to realise the futility of this attitude and to open ourselves up to reality as it is. Nussbaum refers to this process as ‘exposure’ (Nussbaum 2001a, 18–21), which she sees as an essential component for leading an ethical life.

## Moral case deliberation

Moral case deliberation (MCD) is a form of clinical ethics support (Dauwerse 2014; Molewijk et al. 2008; Weidema et al. 2013; Stolper et al. 2010) that has become increasingly popular over the last 15 years. The aim of clinical ethics support is to assist care providers in ethical matters that they encounter in practice. Instead of providing expert advice, new forms of clinical ethics support (such as MCD) aim to provide opportunities that foster moral reflection (Dauwerse 2014, 10). MCD is a structured and methodical dialogue led by a facilitator, in which health care professionals explore a moral issue from a concrete situation in their own realm of experience. The case is brought in by a participant, who was (or is) directly involved themselves. MCD seeks to explore both the factual situation as well as the perceptions and moral perspectives of both the person contributing the case and the other participants.

The purpose of MCD is to have the participants reflect critically on healthcare practice and their associated normative presuppositions, and to improve them wherever possible and desirable. Participants explore their personal moral considerations and share them with one other in the spirit of equality (Weidema et al. 2013, 619). This exchange of experiences facilitates greater mutual understanding and a broadening of perspectives. The primary objective of MCD is not to find a solution to the issue, but rather a ‘fusion of horizons’ among the participants (Gadamer 1960).

The facilitator gives structure and depth to the dialogue by means of a conversation method. This provides perspectives on how to act and thus makes a difference for decisions in medical care. The MCD meetings in which the present case was discussed used the dilemma method (Molewijk and Ahlzen 2011; Stolper et al. 2016). The dilemma method consist of ten steps: 1. Introduction, 2. Presentation of the case, 3.Formulating the moral question and the dilemma, 4. Clarification in order to place oneself in the situation of the case presenter, 5. Analysing the case in terms of perspectives, values and norms, 6. Looking for alternatives, 7. Making an individual choice and making explicit one’s considerations, 8. Dialogical inquiry, 9. Conclusion, 10. Evaluation (Stolper et al. 2016). This method focuses on the dilemma faced by the case contributor, which is described in terms of two mutually exclusive treatment options.^3^ A key feature of the dilemma method is attention to the adverse effects caused by each of the treatment options. This makes it directly compatible with Nussbaum’s concept of tragedy discussed above, which involves two valid ethical claims that cannot both be fulfilled.

## Method

The method used is that of a case study (Yin 2014): a meticulous, in-depth and detailed examination of a series of MCD meetings relating to a tragic situation. The present case concerns not only the patient case that was discussed during the MCDs, but also—and perhaps most importantly—the reflection on the patient case during the MCD meetings. It is an empirical study of what participants understand by tragedy, based on interviews. The case is analysed with qualitative methods, focusing on the views and experiences of the participants (Patton 1990).

### Data collection

In this study, semi-structured interviews were used to interview health care professionals who were both involved in the case and attended the MCD meetings when the case was discussed (N = 10). Twelve of the parties involved were approached for an interview. Of these twelve, one person proved to have had only incidental involvement with the patient and the MCDs, and another had already left the organisation for position elsewhere. A total of eight medical specialists from various fields (a gynaecologist, a gynaecologist/perinatologist, a gynaecologist/sonographer, a neonatologist, a paediatrician/neonatologist, a neurologist, a neuro-oncologist and a neurosurgeon), a GP and a midwife.

The interviewees were asked about what made the case a tragic one in their eyes, and about the role played by MCD in bringing this into relief. The questions asked during the interviews were based on literature studies, participant observation by the researcher during MCDs, and general background discussions with medical ethicists and hospital professionals with MCD experience. All interviews were recorded (with the respondents’ permission), transcribed and anonymised. No ethics approval was required for the study, as no patient treatments were being imposed.

### Analysis

Respondents were given the opportunity to review the text, and revise it where necessary. The interviews were analysed by hand (manual coding) (Saldaña 2013). The transcripts were read and examined sentence by sentence, in search of similarities and differences (initial coding), with sentences being summarised as single words or brief sentences (descriptive coding). Attention was also devoted to any salient words (in vivo coding) or contradictions (vs. coding) apparent in the text. Furthermore, the reflections of the individual researcher(s) during both the interviews and the coding process were noted down (analytic memos). These notes helped to establish links and reveal noteworthy patterns throughout the interviews. All of the codes were subsequently analysed and summarised according to topic (thematic analysis). To guarantee quality, coding was performed by multiple researchers who also acted as peer debriefers throughout the study, and discussion partners for both the design of the study and the results and topics.

### Selection of the case and the associated MCD meetings

The following criteria were applied when searching for a suitable case:

- In view of the research question, the case discussed during the MCDs must include a strong element of tragedy, commensurate with the definition of tragedy given above;
- At least one MCD meeting must have been held regarding the case;
- The parties involved in the case must be traceable and have taken part in the MCD meetings;
- The case and MCD meetings must not have taken place more than one year ago, to ensure that the parties involved can still readily call their experiences and memories to mind.

## The case and the moral case deliberations

The case involves a 38-year old female patient. She has several children. The department’s annual report describes the case as follows: ‘Ten weeks into her pregnancy, the patient was admitted to the neurology department elsewhere due to suspected Cerebro Vasculair Accident (CVA) suggested by loss of strength on the right side and subsequent seizures. A Computer Tomogram (CT)-scan revealed a left-frontal space-occupying lesion. Four weeks later she was referred to a University Hospital, and in the meantime started suffering aphasia and facial paralysis.

The Magnetic Resonanse Imaging (MRI) revealed a progressively growing lesion, and the decision was made to take a brain biopsy. Histopathology revealed an infection consistent with vasculitis. The possibility of a tumour could not be excluded. Following cross-disciplinary consultation, a short course of methylprednisolone was administered to reduce brain oedema and thus relieve symptoms. During the 16th week of pregnancy, a craniotomy was performed to relieve intracranial pressure under a diagnosis of vasculitis. A left-frontal section of bone was removed and an open biopsy taken, which revealed a glioblastoma localised in the leptomeningeal space. Due to the extensiveness, character and multifocality of the tumour, the possibility of further treatment was excluded. The pregnancy had no influence on the prognosis. Despite her aphasia, the patient expressed a clear wish to continue with the pregnancy. Her husband supported this decision.

From the 17th week onwards, the woman was cared for at home under the direction of the general practitioner/midwife in weekly/daily consultation with the neurologists and gynaecologists.

The 20-week ultrasound gave cause to suspect oesophageal atresia in the foetus. The parents declined invasive diagnostics. Although the patient’s clinical condition was deteriorating rapidly, expected time to death remained uncertain since the craniotomy eliminated intracranial pressure as a possible cause.’

The first MCD meeting, facilitated by an ethicist, took place during the 20th week of pregnancy. At this time the patient was still mentally competent. A report of the meeting was drawn up, which formulated the dilemma as follows:

(A) We treat the patient (with a feeding tube/antibiotics to improve the child’s prospects), or
(B) We give no further treatment except for comfort/palliative care.

‘Needless suffering’ was formulated as a negative consequence of option A, and poor prospects for the child in the case of option B. Both alternatives have a direct impact on patient care.

An analysis of the norms and values from the perspectives of each of the parties involved is given below. A ‘value’ represents what is important for a person in the situation at hand, a ‘norm’ formulates the rule of action needed to realize a specific value (Table 1).

**Table 1:** Analysis of norms and values

Perspective	Values	Norms
Patient	Trust	I should trust the doctors
	Lots of children	Now that I am dying, I would like to have this child (even with Down Syndrome)
	Healthy baby	If the baby dies, I can care for it in heaven
	Concern for husband	I have to take care of my husband
Husband	Compassion	I must be there for my wife
	Obedience	I should do what she wants
Patient’s mother	Right to protection (of the unborn child)	I don’t want any discussion
	Willingness to help	My daughter needs help
	Stand up for my daughter	The doctors have to be less clinical
	Distrust	I need to check up on the doctors
Foetus	(No data)	
Neurologists	Patient first	We must not do anything that is not in the patient’s interests
Gynaecologists and paediatrician	Maturity of the child	The intervention limits must be raised to increase prospects for the child
	Support of mother and child	A scenario must be developed
brothers/sisters	(No data)	

Based on the discussion, all participants then considered matters individually and responded to the dilemma formulated above. Each participant also stated which value ‘tipped the balance’, which ones were left unaffected (by not choosing the alternative), and how the damage could be repaired. The individual choices were tabulated and compared with one another, which led to a dialogue among the participants on the similarities and differences, and what could be learned from the viewpoints of others.

By the end of the meeting there was a broad consensus among the participants regarding the course of action to take: option B, i.e. no further treatment except for comfort/palliative care. This was the option chosen to be suggested to the patient for consent. The underlying reason for this choice was poor likelihood of a healthy child. Further treatments would be likely to cause even more harm. To limit the damage, it was agreed to communicate the decision clearly due to the importance of trust in the patient-doctor relationship. They decided to include the patient’s husband and mother in this process, the patient had given consent to their involvement, and to provide them with extensive support. The situation was so exceptional and tragic that it was decided to deliver the decision (and explanations) during a home visit to the patient and her family. A visit to the hospital would be too much for her.

A second MCD meeting was held during the 27th week of pregnancy, led by the same ethicist. A report of this meeting was also drawn up. At the start of the meeting it was announced that the planned home visit did not take place, because the GP had assumed responsibility for communications. The GP was present at this new MCD meeting. The woman’s condition had deteriorated: she now has a large swollen mass on her head, and can only move half of her body. She is bedridden, and can consume liquid foods. She can no longer speak; indicating her understanding is sporadically possible, but is becoming less and less so. At this time she had indicated that her mother should be her representative. Her husband was not talkative, and kept himself in the background. The decision against invasive life-prolonging treatment in the interests of the child is still in force. The situation has changed however, as the child now has a chance of survival if it is born. The new question concerns what to do if the patient’s condition suddenly worsens, presenting an acute threat to the child. Moral choices concerning medical decisions not only have impact on the patient care of the mother, but also on the life of the child.

The interests of the child are now paramount, and any decisions should aim to give the child the best possible chance of survival and quality of life. Consequently, this would mean delaying the birth for as long as possible. One crucial aspect of the child’s prospects is the question of the oesophageal atresia (and the possible complication of Down Syndrome). There are signs that this may be the case. Operating on a child for oesophageal atresia before 32 weeks is difficult, and chances of survival are slim. They decide to perform another ultrasound. This would require the patient to come to the hospital, unless a company can be found that would be willing to provide a portable ultrasound scanner. The sonographers agree to try to organise one.

Now there are two conceivable scenarios. If oesophageal atresia is confirmed, it only makes sense to take action after 32 weeks if there are any complications. Hospital admission for feeding tubes or a C-section is only useful after this point—until then, the policy is to wait. In the absence of oesophageal atresia, the 32-week limit does not apply. The importance of the ultrasound and the two scenarios will need to be thoroughly explained to the family.

If a C-section is required, for example, what should be done if there are any complications (such as haemorrhaging)? Although the woman ultimately has no chance of survival, denying any form of treatment would seem rather extreme. They decide to treat her normally (e.g. via a blood transfusion), and to avoid invasive procedures such as operations or admission to IC. There is a limit regarding what would be beneficial, given her limited life expectancy and quality of life.

Because the ethical issue had not changed significantly since the first meeting, there was no need to carry out a new analysis in terms of norms and values. The discussion revolved primarily around how to apply the previous normative conclusions in light of the new circumstances (improved prospects for the child). The outcome of the MCD meeting was to continue along the lines established during the first meeting: administer no treatment to prolong the woman’s life that could potentially endanger the child. Despite the tragic nature of the situation, this perspective allows a clear line of action to be established that everybody can agree with. Communication with the family remains an important issue.

The department’s annual report describes the conclusion of the case as follows:

(-) A home visit was then made by the gynaecologist, midwife and sonographer from the University Hospital to carry out another detailed ultrasound. This screening, at 27 + 2 weeks, revealed a case of Intra Uterine Foetal Death. The patient died at home that evening. No autopsy was performed.

Some weeks after the patient’s death, a third meeting with the ethicist was organised in order to look back on the events and decisions that were made with those involved. This concluding session was freer in character, and no structure was imposed by the ethicist.

The three meetings were attended by a total of twelve healthcare professionals in varying combinations, ten of whom were interviewed. Three of the ten interviewees had prior experience with MCD. The interviews were conducted 1 year after the case and the MCD proceedings.

## Results

This section seeks to successively answer the three sub-questions formulated above. First we will describe five tragic elements in the case according to the respondents, and then discuss five aspects of moral case deliberation that played a role in helping the tragedy to manifest. Lastly, we give the respondents’ opinions on what is required during moral case deliberations on tragic situations such as this.

### What characterises the tragedy in this case?

Respondents were asked what characterised the tragedy in this case. Five elements were found.

The first element that the respondents believed characterised the tragedy was its *impact*. All respondents state that the case stayed with them. Even after a year had passed, they could still easily call the situation to mind without needing to refer to the medical reports. One of the respondents described the repercussions of the case as follows:

There are some cases that just stay with you, and this is one of them (…). The tragedy of a pregnant woman with both a child in situ and a rapidly progressing malignant process… it leaves its mark on you. It gave me sleepless nights, and (…) the problem was we were always dealing with mother and child, we had to consider both. (Interview 5)

A second element was the *intensely sad* nature of the situation. The respondents called it a ‘sad’ situation for both the mother (who is carrying a child that she will never be able to raise) and for her partner (who will be left with several children). They were also emotionally affected by the situation, and the fact that there were several other children amplified the feeling of sadness:

Yes, absolutely. Of course we were all incredibly consumed by the tragedy of it all. And we… everybody could at least… you know, we could get it off our chest, so to speak. But of course, we all felt, maybe some of us were secretly kind of thinking like, man, the husband, you know. It’s all well and good for her to want the child, but her husband already has all those kids to deal with, and then there could be an extra disabled one, with all the extra care required. What on earth is he supposed to do? (Interview 4)

The third tragic element is the *acceptance* of the inevitable. The inevitability of the mother’s death was of course openly expressed during the MCD sessions. The respondents were ultimately relieved when mother and child died together, giving them a certain peace of mind:

I’m glad things went the way they did, in the end I’m happy she died with her baby inside her, and that they were buried together. It was just like she wanted, so I am at peace with what happened. (Interview 10)

The fourth tragic element revealed by the interviews was *powerlessness*. The case presented an unexpected turn of life events attributable only to bad luck and misfortune, which made those involved feel powerless.

The word actually says it all, right? (long pause) An insurmountable… (long pause) …something ominous with an… inevitable conclusion. Something that… ‘cause it’s tragic, of course. (-) And it’s irrevocable too, there are no winners. It’s the worst thing you can imagine. (-) There’s no way around it, you know? It’s going to happen… powerlessness’. (Interview 7)

The fifth element of tragedy concerns the threat to *human dignity*. The decision of whether or not to provide treatment will affect how the patient will die, and particularly whether she can do so with dignity:

For me, the complex issue was the huge list of possible scenarios due to the combination of the patient’s malignant disease and missing a piece of her skull. (-) And the list only got longer, because all the scenarios we created for the mother also had consequences for the child. So making her feel as comfortable as possible – essentially giving her a… a dignified death in the relatively short term – that of course denies her child the opportunity of being born alive. On the other hand, a barrage of treatments to extend the mother’s life would make her situation more and more undignified… [but] would improve prospects for the child. (Interview 9)

### How did moral case deliberation bring this tragedy into focus?

What role did MCD play in bringing the tragedy of this case to the fore? The interviews revealed five aspects of the role played by moral case deliberation in tragic situations.

The first of these is the fact that MCD *clarifies the dilemma* through the concrete formulation of two treatment options. The dilemma method places the emphasis on conflicting values and interests. The dilemma during the first MCD session was formulated by one of the respondents in the following quote:

During that MCD session (…) the main issue was: what things are important for the mother, and which are important for the child? The real moral component was that any decision to treat the mother and reduce her suffering might do damage to the child. (Interview 6)

Giving comfort to the mother and ceasing treatment means that she will die sooner, but will deny the child the opportunity of being born alive. Conversely, while all treatment to extend the mother’s life increases the prospects for the child, they prolong her suffering.

The second MCD session focused on the child. During the 20th week of pregnancy, there was reason to suspect that the child may have had a serious birth defect. Healthy children with enough bodyweight can be born prematurely, as they are more likely to survive. This child’s chances were slimmer, however, due to the suspected abnormality. The question was also raised as to whether serious trouble should be taken to save a child with a severe disability.

The second aspect concerns the open discussion of the *damage caused*. The dilemma method explicitly defines the damage accompanying certain choices, e.g. exploring the consequences of giving the patient chemotherapy or not. Treating her with chemotherapy would threaten the child’s development, demonstrated by the following quote:

When everything started, it was still quite early in the pregnancy… And so all kinds of things can enter the equation, you know? At one time, I think, the idea was proposed of treating the patient with chemotherapy. Well… of course, that would affect the child’s development. But even at that early stage, she didn’t want to… to terminate the pregnancy. (Interview 3)

Giving nutrition via a feeding tube would also have prolonged her suffering:

Once it became clear what we were dealing with, a whole new set of dilemmas presented themselves. What to do? I still remember very clearly that the patient’s mother came here for an appointment, saying gosh, she’s starting to have trouble eating, shouldn’t we try a feeding tube or something? And those were things that I really did have trouble committing to, because they would actually only prolong her suffering. (Interview 6)

As care professionals, the respondents feel a responsibility to explicitly name the damage during MCDs. Opting for the patient’s desire to bring the child into the world and moving the birth forward would affect the child’s chances of survival. But if it does survive, they run the risk of leaving the father with a disabled child:

She really wanted to carry the baby to term, and her final goal in life was to bring that child into the world. But she had had so much medication for her operation and her brain tumour, and the child just wasn’t growing properly. (-) Were we supposed to take the child out far too soon? (-) Half of all children born under 26 weeks never make it anyway, and those that do survive are severely disabled. Should we really do that to the father, who is all alone in the world and with a family to care for? To lose his wife, and then be left with a disabled child? But fair enough, that’s what she wanted. (Interview 4)

The third aspect is that of *putting oneself in the situation*, which involves the participants concretely imagining what is going on. They see a real picture of a woman lying there, with a tumour growing *out* of her head. The respondents stated that this allowed them to easily feel the tragedy of the situation, which can sometimes evoke memories of earlier, personal experiences, as relayed by the following interviewee:

An important fact to realise is that my mother also died of a brain tumour at her [the patient’s] age, leaving similar-age children behind, so I had a very clear idea of what it was like. It meant… of course you feel emotional, but I was still able to keep a distance, I wasn’t overly affected. Familiarity with the situation meant that I could contribute and that I had something to offer, like what is important for your children, what do you want them to remember, and letting go… (interview10)

The fourth aspect concerns *insight into the perspectives* of the others involved. Because MCD examines the dilemma from a variety of angles, participants can reflect on their own motivations and those of others:

I was very grateful that we always discussed the matter as a large group. Everybody who was involved, the GP came, the neurologist, neurosurgeon, the clinical ethicist. The situation was viewed from all angles. (Interview 4)

The exchange of perspectives within a multidisciplinary setting raises understanding of the situation, and helps create a support base for the ultimate decision to be made in the dilemma. Taking the decision carefully and in consultation with others helps the MCD participants to move forward.

The fifth and final aspect relates to the *weighing up of values*. MCD places the emphasis on moral aspects, whereas treatment plans are drawn up and discussed according to established medical guidelines. The course taken by MCD discussions differs from those of a purely medical nature—the structure and guidance provided by the facilitator in particular help to get to the heart of the matter:

It was much more about the various moral aspects involved, and examining them together in a very structured way. Because moral deliberations are not part of our day-to-day, (…) I found it a very good approach. It brought me a great deal of clarity (…), the heart of the matter (…). I found the structure very helpful, and also the presence of a facilitator with a neutral, objective stance (…). I think all of the specialists would have appreciated it. We do each tend to look at things from our own little corner, after all. (Interview 4)

Making the values and norms explicit that play a part in the dilemma exposes the conflicts, revealing the tragic aspect of the case:

The extra dimension of MCD? Well, because, let’s say, it was about… usually things are pretty clear, a child either has a defect or it doesn’t, and you decide to treat it or you don’t, and when it should be born is all pretty clear, but here there were two significant interests involved that, um, let me put it this way, the interests complicate things, the conflict of interests is more pronounced. Deciding against one thing will put the other at a disadvantage, so to speak. So deciding not to treat the mother will also reduce the prospects for the child. (Interview 1)

### What do people need in tragic situations?

The respondents were asked what it is they need when confronted with a tragic situation. Five elements proved to be important. The discussion of these elements also looks at the question of the extent to which MCD in its current form meets the needs of the participants in tragic situations.

The first point identified by the respondents concerns the *opportunity to share and discuss* the thoughts and feelings elicited among care providers by tragic cases. According to respondents, expressing and sharing the emotions evoked by a tragic situation requires greater attention:

The topic should be more open for discussion, I think. But I also believe that professionals should be trained to deal with it. I mean, aside from the emotions involved and the horrible events surrounding them, that it doesn’t automatically mean that you can no longer do your job as a professional or that you need to take extended time off or whatever, but that you learn how it is (…) possible to live with it and retain sufficient confidence in your own ability to continue working as a professional. I would be in favour of that. (Interview 1)

The interviews revealed that MCD is helpful in tragic situations because it provides the opportunity to discuss *matters that touch people*:

(…) certainly in all of the MCD meetings too, and especially during the final session when we wrapped things up. Because there had been informal communication that she had died, but we didn’t see one another then, and I did find it important to give things a proper conclusion, a fact that came out strongly again during that meeting. And the one who was most deeply involved, that was [midwife’s name]. Because she’s, she can also describe the family really well. She also went to the funeral, and is good at telling how it all went, with a great deal of attention. (-) That’s why she was so touched by it all. (Interview 8)

MCD participants sit in a circle, which facilitates the sharing of experiences. During clinical discussions, the participants are often seated side-by-side, facing a screen showing projections of the case data:

After that, when the scans were available and the diagnosis had become clear, we had a meeting in one of those rooms with… a radiology room I think it was, a really big room with all the test results shown up the front using the projector. But everybody was sitting side-by-side, and we were right up the back, so we were mostly looking at people’s backs, people did turn around… (Interview 10)

The second point is *care for oneself and for each other*. Tragedy has a major impact, and flips a switch in those involved. Especially within the context of an academic hospital, where all of the complex and serious cases from the region converge, and where doctors and nurses therefore see a lot of tragedy.

Firstly, care for oneself is one important aspect of dealing with tragedy:

So, when something like this happens, it’s important for you as a person to have a support network. Of course there are your immediate colleagues, who don’t necessarily need to discuss all the details of the case, but more like gosh, how are you going to process that? (…) That’s the inner circle of course (…). But besides that it’s also very important for people to have lots of extra circles – family and friends – to provide support, like, if it’s something that will be affecting you for longer than the average patient in an emotional sense. If you hit a roadblock or… then do you think that… your professional life will keep going well? Not for long. (Interview 9)

Secondly, it is important to *care for one another*. Tragedy places great demands on those involved, as demonstrated by the following quote from the sonographer:

I went to do the ultrasound, and I was pregnant myself. (…) Everyone really was a little worried about me. I remember that the professor of neurosurgery even gave me a phone call, that was very thoughtful of him. And a week later, during my visit to the clinic, they asked “ And? How did it go?” And: “ It was so brave of you to go do it.” That was really nice I thought. And one of my colleagues also came along with me. She said yeah, you can’t go by yourself. So in that sense there was (…) plenty of support. (Interview 4)

The third point identified as important by the respondents was *need for structure*. The purpose of structure when talking about tragic situations is to prevent participants from getting mired down in the emotional discussions elicited by the tragic case. The facilitator plays a key role in this respect:

The idea was certainly to arrive at a decision according to a schedule. And I think that MCD – especially when facilitated by someone who knows what they’re doing – also means you don’t get bogged down in all manner of emotional or other discussions; it may sound a little clinical, but not staying on task and making a decision… I think it was achieved in a very structured way. (Interview 6)

The structured nature of the MCDs also raised questions. Two of the respondents did not feel supported by the method:

And the pros and cons, that sort of thing you know, it was all forced into a kind of mould, and I thought, I actually thought it was a little unnatural. Those pros and cons, we’re already doing that in our own heads, continually actually. (…) I actually found it a little contrived, the pros and cons, yeah it… And then you even need to sit down and formulate everything. (Interview 5)

A fourth point concerns attention to emotions. One respondent perceives MCD as a ‘rather businesslike discussion’, and believes that discussions of tragic situations should include more opportunities for *emotional reflection*.

I can well imagine that you… that it would be good to be able to discuss certain emotions more easily (…) because it’s a rather businesslike discussion after all, those norms and values. Behind norms and values are always emotions, of course. And that, it might be a good idea I think, to provide that opportunity, from a certain perspective of reflection, so to speak. (Interview 1)

*Humour* is also important for dealing effectively with tragedy:

I think that there should be room for the emotional side of what we do and the cases we encounter, (…) so that includes the humorous aspect. Humour is also very important, which means the other side as well. So it’s, that aspect should be included too. Even hospitals need a bit of normality. Normal people, actually using your ordinary eyes to keep looking at people, who just happen to find themselves in an awkward situation. (Interview 5)

In addition to the necessary attention to emotions, respondents also talked about the importance of *reflecting on one’s own attitude to life*. MCD should target attitudes related to life problems, and contribute to the examination of personal motivating factors:

Yeah, and formulating your view of big life questions. Hard ones… (-) Yeah, life, um, problems that present as a part of life. Like, what is your attitude to them. How do I see them? How critical am I, and why am I critical? What are the important factors? Is it my emotions, my beliefs, is it culture? Is it my ignorance? My insecurity? What, what is it? What motivates me?

A fifth point is the fact that people want to *learn* from the case, particularly with respect to similar future situations. For this reason, the respondents say it is useful that the case was discussed not only during MCD meetings, but also in casuistry discussions with gynaecologists, paediatricians and midwives from the local region:

Yeah, because I think it’s, there’s a valuable learning experience here for doctors in various stages of their training, (-) because what you want to avoid is for this to become a sort of (-) story that’s whispered in the corridors, you know? She’s a very ill patient in a very complex situation, with aspects that you want to put into perspective for all those involved. The story shouldn’t do the rounds at drinks sessions. It’s just, yeah, a very complex medical problem. And the thing you notice about trainee doctors is precisely the emotionally charged aspect, which of course means that they want to discuss it with everybody they believe can help them, and I think it should be given a proper forum, not like “ Did you hear about that patient? Well listen to this…”, no. But holding in-depth discussions with those around you in order to find a way for yourself to deal with things and to make decisions and so on, I think that’s the way to get the greatest learning benefit out of the situation. (Interview 9)

## Discussion

The text above constitutes an investigation into the possible contribution made by moral case deliberation in dealing with tragic situations. The interviews revealed five key elements of tragedy: the ‘lingering’ nature of the case, the experience of intense sadness, acceptance, powerlessness and the threat to human dignity. Tragedy also proved to be evinced by the following five aspects during MCD: formulating the dilemma, explicitly stating the damage, putting oneself into the situation, insight into others’ perspectives and weighing up different values. Lastly, five points for attention were highlighted for dealing with tragedy, namely: sharing with one another, care for oneself and for each other, a need for structure, talking explicitly about emotions, and learning from the situation.

The results have shown that a key feature of the tragic situation in the present case study is its tenacity in the memories of the healthcare professionals. This element of tragedy is addressed from the perspective of the ethics consultant in the book titled ‘Cases that haunt us’, which states that tragic cases ‘(…) linger in the memory’ (Ford and Dudzinski 2008, XVIII). Those involved learn that ‘They should be conscious that, often enough, they are working around (-) irreconcilable conflict.’ (p. XVIII).

The results may give rise to the question whether there is a difference between tragedy and moral distress. Although both may involve similar experiences, the crucial difference is that in the case of moral distress the healthcare professional knows the right action, but is prevented through external or internal reasons to act in accordance (Pauly et al. 2012), whereas in the case of tragedy there is no really good choice, since both alternatives come with moral damage.

The other four characteristic elements of tragedy (intense sadness, acceptance, powerlessness and human dignity) are all ripe with existential elements (Browall et al. 2014; Alma 2005; Kenny 2006). The existential aspects of tragedy are all linked to the inherent disconnect between what humans believe they can actively bring about (agency) and external events, or what is fixed or coincidental (contingency) (Nussbaum 2001a). Health, friendship, love and possessions are all valuable things, but they also render existence vulnerable. Many things in life cannot be managed or controlled. Sometimes there is no option other than to live through and endure the situation.

The results show that MCD helps people to deal with contingency. MCD as a specific from of Clinical Ethics Support (CES) differs from Clinical Ethics Consultation (CEC). In CEC, the ethicist acts as a consultant, who reads the medical files, speaks with those involved, searches for a solution given the situation all heard. (Aulisio et al. 2000; Tarzian and ASBH Core Competencies Update Taskforce 2013) The moderator in MCD only facilitates the process. In MCD the focus is on dialogue and reflection among the participants. Because the interest of the patient is paramount, it is recommended that the patient or his/her representative is present during the MCD meeting. This was not possible in this case because of the severe illness of the patient.

Reflection happens first of all through the formulation of the dilemma and explicitly stating the damage, demonstrating that there is no simple solution in terms of agency. The reflection and dialogue during MCD supports healthcare professionals in the difficult decisions they face. In tragic situations, people need to accept that the ultimate solution will always also cause some damage (contingency), which poses a threat to one’s morality.

This is also addressed by putting oneself in the situation, a process that is aimed at helping people to visualise the situation, and which brings back memories of personal experiences among the respondents. Visual images lie at the core of recollections of traumatic events (Janoff-Bulman 1992, 55). Personal memories of confrontations with suffering and death provide a basis for reflecting on one’s own values and life questions. Although healthcare professionals are confronted with patient suffering and death on a daily basis, reflection on life questions is not a part of medical training programmes. Visualising tragedy is in this sense like entering uncharted territory, and can be identified by the term ‘deterritorialisation’ (Deleuze and Guattari 2003, 381). Moral case deliberation can open these images up for discussion.

According to Liaschenko et al. (2006), the link to one’s own moral experience is crucial for adequately dealing with tragic situations in health care. When discussing tragic casuistry, medical students are often distanced from the case: it is analysed and ‘solved’ through the application of principles, and students are allocated the role of ‘observer’. Liaschenko et al. point out that focusing on a solution (an attitude that, according to Nussbaum, is evidence of agency) distracts from the search for a moral stance. In education, being open to one’s own doubts and learning from each other is key.

The results of this study show that, unlike standard discussions about patients, MCD prompts participants to formulate and share personal experiences with one another and thus helps to create a shared perception of the situation as both tragic and contingent.

The respondents identify various needs when dealing with tragedy. First and foremost is the need to share with one another, for which MCD provides a solid basis. In MCD sharing and recognizing each other’s struggles and concerns creates a mutual bond. Analyzing values may contribute to mutual understanding (Molewijk et al. 2011b). Respondents also indicate a need to care for themselves and for each other. This care is not provided during MCDs themselves, but participating in MCDs fosters a climate of support. The exploration of what people need in tragic situations is also addressed in other disciplines (Renzenbrink 2011; Collins and Long 2003; Janoff-Bulman 1992; Benson and Magraith 2005). These studies show the importance of colleagues who attend to each other’s wellbeing and the importance of talking about thoughts and feelings in a safe environment (Collins and Long 2003; Johnson et al. 2004). In Balint group work for health care professionals the doctor-patient relationship is discussed and peer support is provided. Participation in Balint group work has the potential to prevent fatigue and burnout in participants (www.americanbalintsociety.org). Yet, MCD is different from psychological support or Balint group work, as ‘addressing emotions in MCD focuses on a deeper conceptual insight and a personal learning process regarding the moral issue at stake’ (Molewijk et al. 2011b).

Thirdly, respondents stated a need for structure. Respondents value the structured approach of moral case deliberation, as it gives depth to the dialogue. Structure fosters insight into values and norms, and is important for moral learning. However some respondents noted that the structure must not take on too much of a ‘schoolroom’ character—the conversation should entail more than mechanically ‘filling in the blanks’ between pre-defined elements. The structure should encourage reflection, inquiry and dialogue among the participants (Weidema et al. 2013). MCD aims at moral learning. The facilitator plays an important role in the learning process of the participants, by assisting them in focusing on and exploring moral aspect of the case (Stolper et al. 2016).

Fourthly, moral case deliberation should devote attention to the participants’ emotions and ethical attitude to life. Emotions are evidence of norms and values (Nussbaum 2001b), and of the things that matter in life. To date, the role of emotions in moral case deliberation has been discussed very little in the literature (for exceptions, see Molewijk et al. 2011a, b). The above-mentioned disconnect between agency and contingency means that emotions are of key importance. Because emotional reflection during MCD highlights values that help to steer the course of action to be taken, aspects of contingency can shed light on what kind of agency is important. Contingency and agency remain at odds with one another, however reflecting on contingency can help to clarify what is at stake when taking action, providing an indication of whether action is required, and if so, what kind. Humour, which the respondents mention as important even in this case, plays an important role in cases of tragedy (Taels 2008; Collins and Long 2003). It helped the participants to see the patient not as an object of medical treatment, but as a subject, a human being needing help. In addition to emotional reflection, the results also suggest reflection on one’s own attitude to life as a point for attention. Tragedy means being confronted with life questions, a situation in which cultural and religious aspects can play a role.

One final point relates to learning from the experience of a tragedy. This learning has both a medical and an ethical dimension. Deriving learning from the situation has a two-pronged effect: it helps to acknowledge and accept the contingency, and prompts consideration of whether the insights gained can be used to improve future decisions. This way, moral case deliberation offers a platform for moral learning through investigating the relationship between contingency (powerlessness) and agency (responsibility). It teaches people to explore the values hidden in the contingency, thus facilitating targeted decision-making.

## Conclusion

Tragedy concerns essential aspects of life, such as suffering and death. It puts life into perspective, and brings an awareness of what is truly important. As Janoff-Bulmann says: ‘They have made their peace with the inevitable shortcomings of our existence and have a new appreciation of life and a realization of what is really important.’ (Janoff-Bulman 1992, 175). In addition to the emotional burden on those involved, tragic situations also demand attention to existential ideas in order to deal with tragedy as it is.

Moral case deliberation facilitates sharing the experience of tragedy, and the ability to manage the five elements raised by tragedy. MCD helps to define the contingency in tragic situations. Formulating a dilemma, explicitly stating the damage caused, insight into others’ perspectives, putting oneself in the situation and visualisation prove to be important tools for gaining an understanding of personal norms and values and searching for a moral stance. Tragic situations present a combination of contingency and agency. MCD in tragic situations could be improved through an awareness of not only the medical and moral, but also the emotional and existential concerns present in the case and during the MCD sessions. Effective incorporation of these concerns in MCD and explicit reflection on the contingency aspect of tragedy will improve participants’ ability to accept and morally learn from the situation, and to forge a path through unknown territory. In this way, moral case deliberation can help participants come to terms with the dilemmas they experience when having to work around an irreconcilable conflict.

## Footnotes

1 Recent research that discusses the gravity of forced decision-making: ‘Sharing the burden of deciding: How physicians and parents make end-of-life decisions’ (Vos-Broerse 2015).

2 In tragic cases, Fredriksen speaks not in terms of guilt, but in terms of responsibility. ‘Professionals do not have to accept responsibility in the sense of culpability—in the sense that they misjudged the situation and should have acted differently. But they must accept responsibility(-)’ (Fredriksen 2006, 452).

3 As opposed to the Socratic Dialogue, which uses a conceptual question as a starting point (Kessels et al. 2006, 2009).
